# Retraction: *Coxiella burnetii* in Humans and Ticks in Rural Senegal

**DOI:** 10.1371/journal.pntd.0014195

**Published:** 2026-04-09

**Authors:** The PLOS Neglected Tropical Diseases Editors

The *PLOS Neglected Tropical Diseases* Editors retract this article [1,2] due to concerns about compliance with the PLOS Human Subjects Research policy.

The Materials and Methods section in [1] reports that the study involved blood samples collected in 2008 and breast milk samples collected in 2009, as well as saliva and stool samples (collection period not reported). The ethics approval statement reported in this article states that the project was approved by the National Ethics Committee of Senegal and Local Ethics Committee (Marseille, France), but does not report approval reference numbers.

A representative of the Aix-Marseille Université Ethics Committee stated that the institutional investigation into the ethics concerns concluded this article meets ethical standards. They stated that the area where these samples were collected is subject to a long-term longitudinal study that started in 1990, and that a dispensary was set up with the aim of studying malaria whilst also providing care to the local population. The representative states that during the longitudinal study various research teams intervened in these villages, in agreement with the local authorities, to carry out follow-up studies and to set up a serological bank from which some of the samples analyzed in this study originated. The representative provided the following ethics approval documents for editorial review:

- Ethics approval document N°09–023, issued on December 09, 2009 by the Comité d’Ethique de l’IRF48 for a study titled “*Recherche de Coxiella burnetii, l’agent de la fièvre Q au Sénégal*”.
- Ethics approval document N°969/MSPM/DS/DER, issued in September 2006 by the Ministère de la Santé et de la Prévention Médicale of the République de Sénégal for a study titled “*Etude de l’histoire naturelle du paludisme: protocole d’étude mené dans les villages de DIELMO et NDIOP arrondissement de Toubacouta Région de Fatick/SENEGAL*”.
- An ethics approval document without a reference number, issued on August 17, 1999 by the Comité d’Ethique de l’Institut Pasteur de Dakar for a study titled “*Etude randomisée en double aveugle de la tolérabilité et de l’efficacité de l’Astesunate plus Amodiaquine comparée à l’Amodiaquine plus placébo pour le traitement des accès de paludisme non compliqués à Plasmodium falciparum à Mlomp (Casamance), Sénégal*”.
- A document without a reference number issued on the January 06, 1994 by the Comité Consultatif d’Ethique Faculté de Médecine et de Parmacie UCAD – Dakar. This document appears to certify that biomedical and epidemiological studies involving villages from Dielmo and Ndiop are conducted in accordance with certain principles.

PLOS reviewed the documentation provided by the institution and concluded that the documents did not fully resolve the journal’s concerns. Specifically,

- The ethics approval document N°09–023 was not issued by a local ethics body in Senegal. PLOS’ Human Subjects Research policy requires authors to obtain prospective ethics approval from local ethics bodies for studies involving human subjects or samples collected from human subjects. In addition, PLOS identified potential competing interests between the committee that granted the ethics approval and one or more of the article’s authors.
- The ethics approval document N°969/MSPM/DS/DER and the document issued in 1999 provide approval for studies into the natural history and treatment of malaria respectively. These documents do not report approval for the reuse of samples collected during these studies for secondary analysis as part of a separate study described in [1].
- The document issued in 1994 only comments on the collection and handling of blood samples. The document does not report the title of a specific study nor does it appear to grant ethics approval for these studies. Furthermore, PLOS considers it unlikely that an approval issued in 1994 would be applicable for sample collection carried out in 2008 and 2009.

CSocolovschi and CSokhna did not agree with the retraction. OM, FF, GD, HB, JFM, JFT, and DR either did not respond directly or could not be reached.
